# Digitalis in Heart Disease

**Published:** 1861-01

**Authors:** 


*Digitalis in Heart-Disease.*—Dr. Germain thus resumes an able monograph on the action of Digitalis :

I. Notwithstanding the opinion of Sanders, which, moreover, is in contradiction with all observations before and after him, Digitalis lessens the frequency of the heart contractions.

II. Nothing proves that it weakens the *force* of the heart's contractions ; while theory, and physiological experiments, and my own experience, prove that one of its secondary effects in narrowing of the heart's orifices, is to augment it—consequently there is no danger in giving it in cases where the heart's energy appears diminished.

III. The frequency of the heart's contractions in the case of narrowing of the orifices of the heart, preventing the organ from returning to a normal activity, and keeping up the disorder of the circulation, and digitalis having the power of diminishing the frequency of the contractions, it is not necessary to invoke another mode of action to explain the amelioration which follows, in this case, the administration of this plant.

IV. Nothing is found in the writings of authors to prove that Digitalis enjoys diuretic properties, that this reputation given it by Withering appears to have been accepted without discussion by all those who followed him.

V. It is true that in the organic affections of the heart where the employment of Digitalis produces an amelioration of the circulation it often produces abundant diuresis, but this diuresis is but a secondary effect, from the return of the circulation to its normal state.

VI. All authors are unanimous in recognizing the powerful action of Digitalis on the stomach. In very small doses it stimulates the appetite, but in the doses in which it acts upon the heart, it produces anorexia, even nausea; and may become a dangerous cause of dyspepsia in the greater number of cases. —(*Gazette Hebdomadaire*, *November.*)

